# circRNA-Mediated Inhibin–Activin Balance Regulation in Ovarian Granulosa Cell Apoptosis and Follicular Atresia

**DOI:** 10.3390/ijms22179113

**Published:** 2021-08-24

**Authors:** Mengnan Ma, Huiming Wang, Yi Zhang, Jinbi Zhang, Jingge Liu, Zengxiang Pan

**Affiliations:** 1 College of Animal Science and Technology, Nanjing Agriculture University, Nanjing 210095, China; 2018105019@njau.edu.cn (M.M.); 2019105085@njau.edu.cn (H.W.); 2020105023@stu.njau.edu.cn (Y.Z.); 2 College of Animal Science and Food Engineering, Jinling Institute of Technology, Nanjing 211169, China; zhangjinbi@jit.edu.cn (J.Z.); liujingge@jit.edu.cn (J.L.)

**Keywords:** granulosa cell apoptosis, circRNA, miRNA, *INHA*, *INHBA*, inhibin, activin

## Abstract

Ovarian granulosa cells (GC) play an essential role in the development and atresia of follicles. Emerging studies suggest that non-coding RNAs are involved in the regulation of GC apoptosis. Here, we aimed to analyze the function of ssc-circINHA-001, coded by the first exon of the inhibin subunit α gene (*INHA*), in resisting GC apoptosis and follicular atresia by enhancing the expression of the inhibin subunit β A (*INHBA*) through a cluster of miRNAs. A higher expression of ssc-circINHA-001 in healthy follicles compared to early atretic follicles was detected by qRT-PCR. Its circular structure was confirmed by RNase R treatment and reversed PCR. The function of ssc-circINHA-001 in GC resistance to apoptosis was detected by in vitro transfection of its si-RNA. Furthermore, the dual-luciferase reporter assay suggested that ssc-circINHA-001 adsorbed three miRNAs, termed miR-214-5p, miR-7144-3p, and miR-9830-5p, which share the common target *INHBA*. A low expression of ssc-circINHA-001 increased the levels of the free miRNAs, inhibited *INHBA* expression, and thus raised GCs apoptosis through a shift from the secretion of activin to that of inhibin. Our study demonstrated the existence of a circRNA–microRNAs–*INHBA* regulatory axis in follicular GC apoptosis and provides insight into the relationship between circRNA function and its coding gene in inhibin/activin balance and ovarian physiological functions.

## 1. Introduction

The quantity of ovarian follicles determines the reproductive rate of livestock [1]. Ovarian granulosa cell (GC) apoptosis is a physiological phenomenon that occurs during all stages of follicular development. Research has demonstrated that the percentage of apoptotic GCs increases significantly with the progression of follicular atresia [2].

Inhibin and activin belong to the growth factor-beta (TGF-β) family of cytokines and play contrasting roles in regulating follicle-stimulating hormone (FSH) secretion, whereby inhibin inhibits and activin activates FSH secretion. Inhibin A and B are heterodimers comprising an α- and a β(A/B)-subunit, respectively. In other words, inhibin A and B arise from three gene products, including the α-subunit gene (*INHA*), the βA-subunit gene (*INHBA*), and the βB-subunit gene (*INHBB*). Activins are homodimers or heterodimers of only the β subunits (βA, βB, βC, βD, and βE). Inhibins and activins counteract each other, and the balance between the amounts of α- and β-subunits will determine how much inhibin versus activin is formed, e.g., increased levels of α-subunit will favor the formation of inhibin, while increased levels of β-subunit will favor the formation of activin [3]. As TGF-β family members, the posttranscriptional regulation of α- and β-subunits expression is largely unknown.

Among non-coding RNAs (ncRNAs), microRNA (miRNA) is the most studied and has been widely reported to participate in follicle development and atresia regulation [4]. Our previous study identified different miRNA profiles in porcine healthy follicles (HF) compared to early atretic follicles (AF) [5]. Furthermore, circular RNA (circRNA) is a new type of ncRNA produced from a precursor mRNA through back-splicing of exons. circRNAs are located in the nucleus or cytoplasm and regulate gene expression through transcriptional (e.g., interaction with RNA-binding protein) or posttranscriptional pathways (e.g., adsorption of miRNAs) [6]. An increasing number of studies have pointed out that circRNA has a significant role in cell differentiation, development, and dysfunction [7,8]. Our previous study showed an abundant distribution of circRNAs in porcine follicles, and the differential expression in HF and AF suggested a possible regulatory function of circRNAs during atresia [9].

In this study, we reported that a newly identified circRNA, ssc-circINHA-001, coded by the *INHA* gene, guaranteed *INHBA* expression by sponging miR-214-5p, miR-7144-3p, and miR-9830-5p, thus further inducing GC resistance to apoptosis and follicular atresia. Our study demonstrated a circINHA–microRNAs–*INHBA* regulatory pathway in GC apoptosis and increases knowledge of the balanced regulation of inhibin A formation and the relationship between the circRNA coding gene and its downstream target gene in ovarian physiological functions.

## 2. Results

### 2.1. Identification and Validation of ssc-circINHA-001

**Figure 1:**
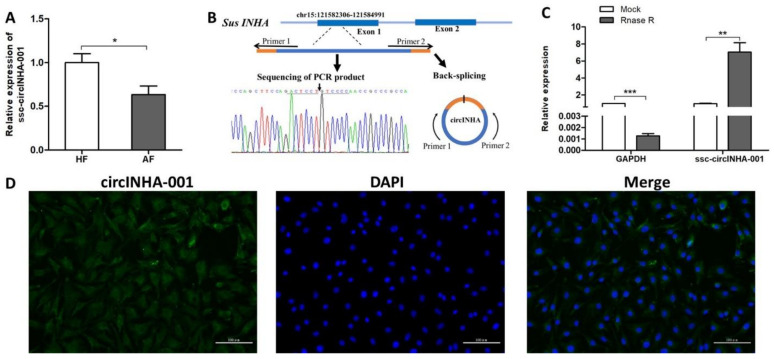
Identification and validation of ssc-circINHA-001. (**A**) Differential expression of ssc-circINHA-001 in HF and AF detected by qRT-PCR (*n* = 6); (**B**) Sketch of the structure of ssc-circINHA-001, which is generated from the first exon region of the INHA gene via back-splicing; (**C**) ssc-circINHA-001 expression after RNase R digestion. GAPDH was used as a control; (**D**) localization of ssc-circINHA-001 in GCs detected by FISH. ssc-circINHA-001 was labelled with green fluorescence, and the nuclei were stained with DAPI (blue). Scale bar: 100 μm. Data are expressed as the mean ± SEM. * *p* < 0.05, ** *p* < 0.01, *** *p* < 0.001.

Our previous deep circRNA sequencing analysis showed that the expression level of ssc-circINHA-001, a candidate circRNA produced from the first exon (5′UTR) region of the *INHA* gene, decreased during follicular atresia [9]. To further elucidate the role of this circRNA in follicular atresia, the expression levels of ssc-circINHA-001 in HF and AF were measured by qRT-PCR, and a significant reduction was observed in AF (Figure 1A). The circular structure of ssc-circINHA-001 was verified by amplification with divergent primers, followed by Sanger sequencing. The result confirmed that ssc-circINHA-001 was formed by reverse splicing of the first exon region of INHA mRNA precursor (Figure 1B). As RNase R resistance is one of the key characteristics of circRNAs, the stability of ssc-circINHA-001 was verified by qPCR after RNase R digestion. A dramatically reduced GAPDH expression level proved the efficiency of digestion. However, an increased ssc-circINHA-001 level was detected, demonstrating that ssc-circINHA-001 was resistant to RNase R digestion and structurally stable (Figure 1C). Furthermore, the distribution of ssc-circINHA-001 in GCs was detected by FISH, and the results indicated that ssc-circINHA-001 was mainly distributed in the cytoplasm of GCs (Figure 1D). These results revealed the existence and circular structure of ssc-circINHA-001, which might play a role in porcine follicular atresia.

### 2.2. ssc-circINHA-001 Inhibits GC Apoptosis 

**Figure 2:**
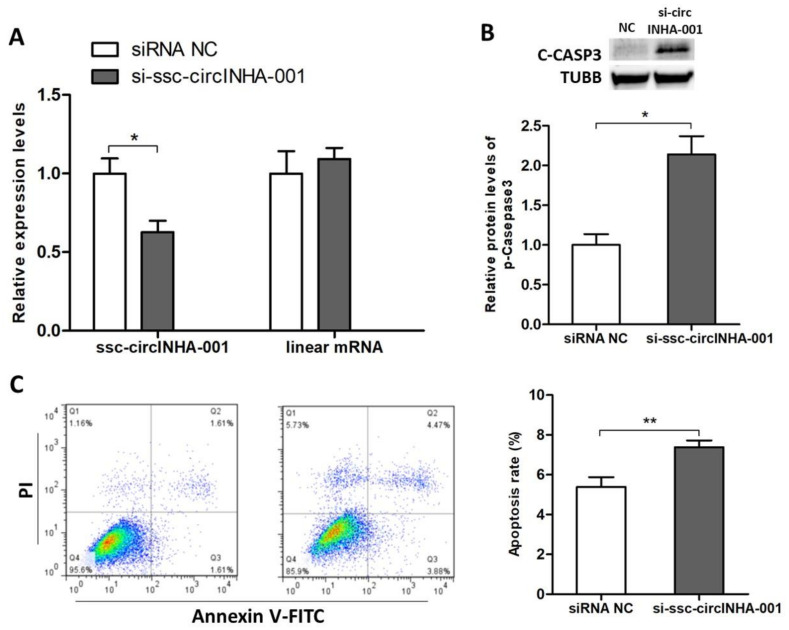
ssc-circINHA-001 inhibits apoptosis of GCs. (**A**) Expression of ssc-circINHA-001 and its corresponding linear mRNA in GCs treated with siRNA control (NC) or si-ssc-circINHA-001 RNA detected by qRT-PCR; (**B**) Protein levels of cleaved CASP3 (C-CASP3) analyzed by Western blot; (**C**) Apoptosis rate of GCs examined by the Annexin V-FITC/PI staining assay using flow cytometry. Data are expressed as the mean ± SEM of three experiments. * *p* < 0.05, ** *p* < 0.01.

To further explore the role of ssc-circINHA-001 in the process of GC apoptosis, a specific ssc-circINHA-001 siRNA, si-ssc-circINHA-001, was designed and synthesized. The transfection of si-ssc-circINHA-001 significantly knocked down ssc-circINHA-001 but had no effect on linear INHA mRNA expression (Figure 2A). WB detection of cleaved caspase 3 (C-CASP3) showed a significant increase after the knockdown (Figure 2B), and the FACS results suggested that ssc-circINHA-001 knockdown increased GC apoptosis rate (Figure 2C). These results indicated that ssc-circINHA-001 had a positive effect on GC survival and inhibited GC apoptosis.

### 2.3. ssc-circINHA-001 is a Common Target of miR-214-5p/miR-7144-3p/miR-9830-5p 

**Figure 3:**
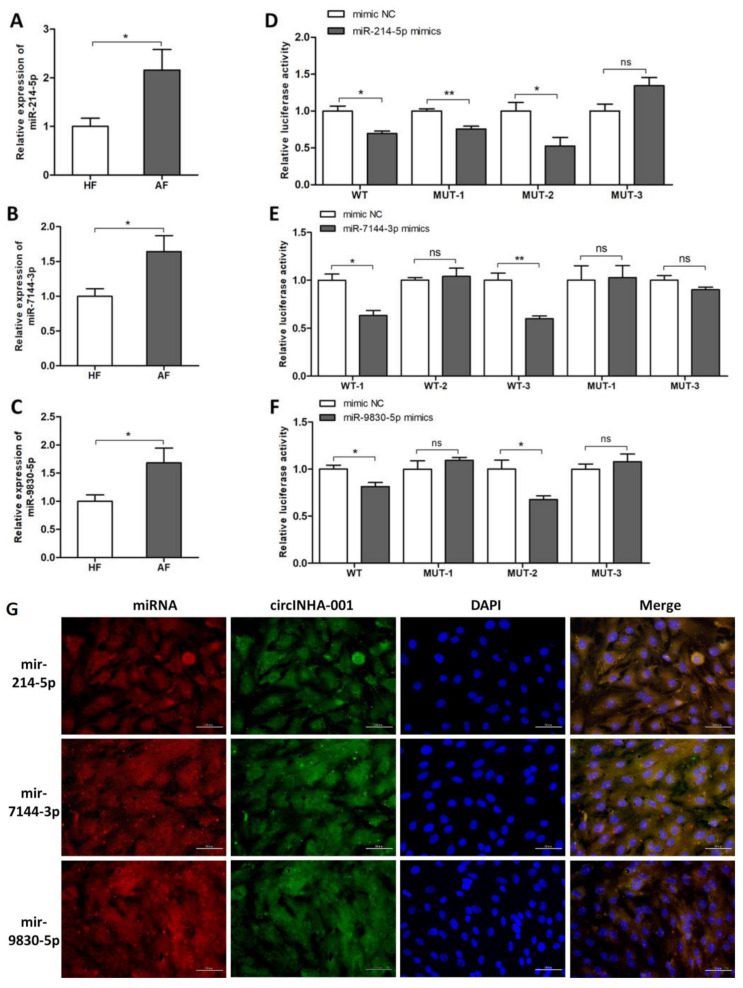
ssc-circINHA-001 is a common target of miR-214-5p/miR-7144-3p/miR-9830-5p. (**A**–**C**). Differentially expressed miR-214-5p, miR-7144-3p, and miR-9830-5p in HF and AF detected by qRT-PCR (*n* = 8); (**D**–**F**). Confirmation of binding sites of miR-214-5p (two sites), miR-7144-3p (three sites), and miR-9830-5p (two sites) on ssc-circINHA-001 assessed by luciferase reporter assay; (**G**). localization of mir214-5p/mir7144-3p/mir9830-5p (labelled by green fluorescence) and ssc-circINHA-001 (labelled by red fluorescence) in GCs detected by FISH; the nuclei were stained with DAPI (blue). Scale bar: 50 μm. Data are expressed as the mean ± SEM of three experiments. * *p* < 0.05, ** *p* < 0.01.

According to our previous miRNA-seq and circRNA-seq results [9], a bioinformatic analysis carried out by Bibiserv (Accessed on 18 June 2020, https://bibiserv.cebitec.uni-bielefeld.de/) predicted interactions between ssc-circINHA-001 and several microRNAs, including miR-214-5p, miR-7144-3p, and miR-9830-5p, during follicular atresia (Supplementary Figure S1). The expression levels of the three miRNAs were confirmed by qRT-PCR and were significantly higher in AF than in HF (Figure 3A–C). To prove the complementary binding between each miRNA and ssc-circINHA-001, dual-luciferase activity assays were performed to verify the binding sites of the three miRNAs on ssc-circINHA-001. Two direct binding sites of miR-214-5p and miR-9830-5p, as well as three binding sites of miR-7144-3p, were proved in ssc-circINHA-001 (Figure 3D–F). Subsequently, FISH assays proved that the cellular location of ssc-circINHA-001 overlaps with the distribution of miR-214-5p, miR-7144-3p, and miR-9830-5p in GCs (Figure 3G). The above results confirmed that ssc-circINHA-001 could be a sponge for miR-214-5p, miR-7144-3p, and miR-9830-5p.

### 2.4. miR-214-5p/miR-7144-3p/miR-9830-5p Enhanced GC Apoptosis 

**Figure 4:**
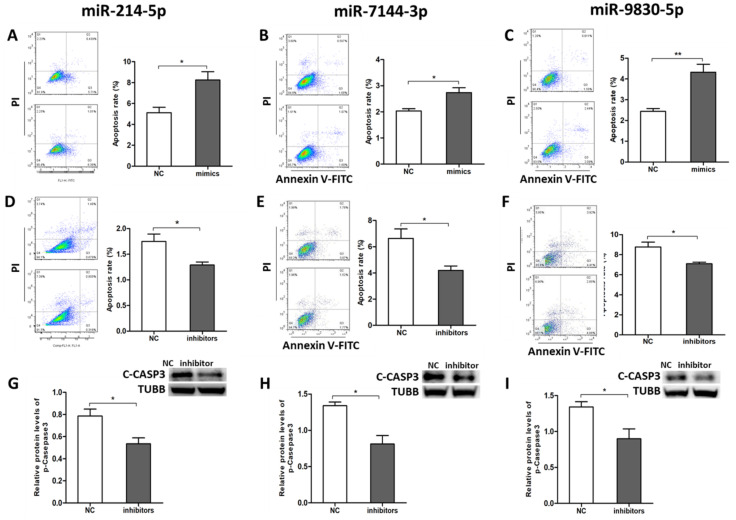
miR-214-5p, miR-7144-3p and miR-9830-5p enhanced GC apoptosis. (**A**–**C**) The apoptosis rate of GCs affected by mimics of mir214-5p, mir7144-3p, and mir9830-5p, respectively, was examined by the Annexin V-FITC/PI staining assay using flow cytometry; (**D**–**F**) The apoptosis rate of GCs affected by inhibitors of mir214-5p, mir7144-3p, and mir9830-5p, respectively, was examined by the Annexin V-FITC/PI staining assay using flow cytometry; (**G**–**I**) The protein level of cleaved CASP3 was affected by inhibitors of mir214-5p, mir7144-3p, and mir9830-5p, respectively, as shown by Western blot analysis. Data are expressed as the mean ± SEM of three experiments. * *p* < 0.05, ** *p* < 0.01.

To further determine the roles of miR-214-5p, miR-7144-3p, and miR-9830-5p in pGCs apoptosis, corresponding miRNA mimics/inhibitors were synthesized for gain- and loss-of-function analysis. FACS detection demonstrated that the addition of mimics of miR-214-5p (Figure 4A), miR-7144-3p (Figure 4B), and miR-9830-5p (Figure 4C) significantly increased GC apoptosis rate. By contrast, inhibitors of these miRNAs significantly decreased GC apoptosis rate (Figure 4D–F). The protein levels of C-CASP3 also showed a significant decrease after treatment with the inhibitors (Figure G–I). These results indicated that miR-214-5p/miR-7144-3p/miR-9830-5p promoted GC apoptosis and hampered GC survival.

### 2.5. ssc-circINHA-001 Affecst GC Apoptosis through miR-214-5p/miR-7144-3p/miR-9830-5p

**Figure 5:**
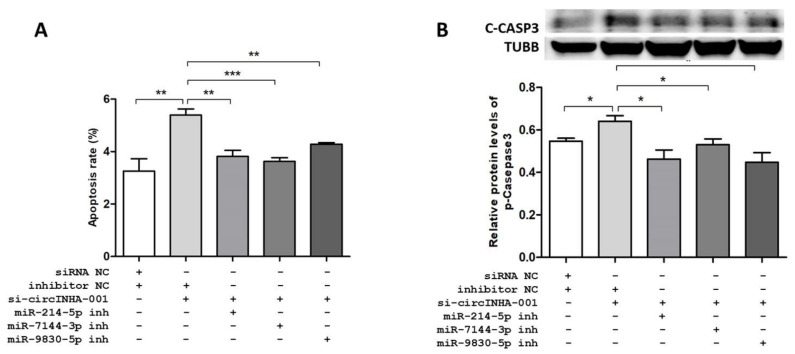
ssc-circINHA-001 affects GC apoptosis through miR-214-5p/miR-7144-3p/miR-9830-5p. (**A**) Apoptosis rate of GCs affected by co-transfection of si-circINHA-001 and inhibitors of mir214-5p, mir7144-3p, and mir9830-5p, examined by the Annexin V-FITC/PI staining assay using flow cytometry; (**B**) protein level of cleaved CASP3 affected by co-transfection of si-circINHA-001 and inhibitors of mir214-5p, mir7144-3p, and mir9830-5p, analyzed by Western blot. Data are expressed as the mean ± SEM of three experiments. * *p* < 0.05, ** *p* < 0.01, *** *p* < 0.001.

To prove the connection among ssc-circINHA-001, miR-214-5p/miR-7144-3p/miR-9830-5p, and GC apoptosis, co-transfection of si-ssc-circINHA-001 and inhibitors of miR-214-5p, miR-7144-3p, and miR-9830-5p, respectively, was performed. The FACS results showed that the raised apoptosis rate, caused by si-ssc-circINHA-001, could be reversed by co-transfection with miRNA inhibitors (Figure 5A and Figure S1). The WB assay showed similar trends, where co-transfection with miR-214-5p, miR-7144-3p, and miR-9830-5p inhibitors reduced the raised C-CASP3 levels due to ssc-circINHA-001knockdown (Figure 5B). These findings indicated that ssc-circINHA-001 suppressed GC apoptosis via CTGF acting as a competing endogenous RNA (ceRNA) for miR-214-5p/miR-7144-3p/miR-9830-5p.

### 2.6. INHBA Regulates Follicular Atresia and GC Apoptosis and Is Targeted by miR-214-5p/miR-7144-3p/miR-9830-5p

**Figure 6:**
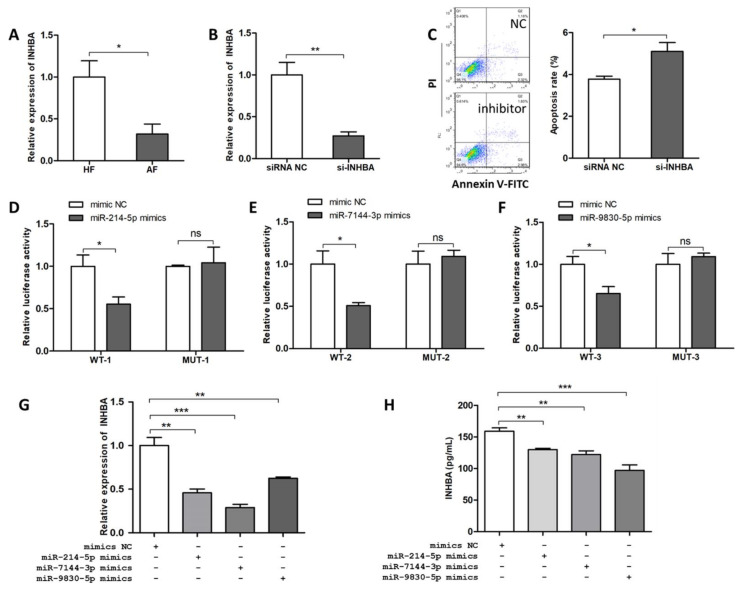
INHBA regulates follicular atresia and GC apoptosis and is targeted by miR-214-5p/miR-7144-3p/miR-9830-5p. (**A**) differential expression of *INHBA* mRNA in HF and AF detected by qRT-PCR (*n* = 7); (**B**) detection of the interference efficiency of si-INHBA; (**C**) effect of si-INHBA transfection on pGCs apoptosis; (**D**–**F**) miRNAs bind to *INHBA* 3′-UTR, as confirmed by the luciferase reporter assay; (**G**) expression level of *INHBA* after miR-214-5p, miR-7144-3p, and miR-9830-5p overexpression in GCs detected by qRT-PCR; (**H**) protein expression level of INHBA after miR-214-5p, miR-7144-3p, and miR-9830-5p overexpression in the culture of GCs, detected by ELISA. Data are expressed as the mean ± SEM of three experiments. * *p* < 0.05, ** *p* < 0.01, *** *p* < 0.001.

Three bioinformatic software, PITA [10], Miranda [11], TargetSpy [12] were adopted to predict the potential target genes of miR-214-5p, miR-7144-3p, and miR-9830-5p, and INHBA was highlighted. To explore the possible function of *INHBA*, the mRNA expression levels of *INHBA* in healthy and atretic follicles were detected by qRT-PCR. The results showed that the *INHBA* level was significantly lower in AF than in HF (Figure 6A). We then synthesized the siRNA of *INHBA* (si-INHBA) to identify the role of *INHBA* in GC apoptosis. The results showed that si-INHBA significantly reduced *INHBA* mRNA levels (Figure 6B), and FACS showed that si-*INHBA* significantly increased GC apoptosis rate (Figure 6C), which indicated that *INHBA* played a role in GC survival.

To prove the binding of three miRNAs to the target INHBA, dual-luciferase activity assays were applied to verify the binding sites of miR-214-5p, miR-7144-3p, and miR-9830-5p at positions 731~737 nt, 239~250 nt, and 605~613 nt, respectively, on *INHBA* 3′ UTR (Figure 6D–F). According to the results, luciferase activity decreased significantly after transfection of the miRNA mimics in wild-type vectors. In contrast, no significant changes were observed with mutant vectors. These observations indicated that *INHBA* is the direct target of miR-214-5p, miR-7144-3p, and miR-9830-5. Subsequently, *INHBA* mRNA levels and secreted INHBA in the culture of GCs were detected by qRT-PCR (Figure 6G) and ELISA (Figure 6H), respectively, after the treatment with the three miRNA mimics. The results showed that both mRNA levels and secreted protein levels of INHBA were significantly decreased after miR-214-5p, miR-7144-3p, and miR-9830-5p overexpression. In conclusion, miR-214-5p, miR-7144-3p, and miR-9830-5p targeted *INHBA* to inhibit its expression and biological function.

## 3. Discussion

With the development of high-throughput sequencing technology, our knowledge of circRNA has rapidly improved. Compared with the other two popular ncRNA groups, miRNA and lncRNA, circRNA is produced by reverse splicing and characterized by covalently closed-loop structures, and thus is more stable and is widely distributed in animal tissues [13,14]. In recent years, circRNAs have been reported as essential regulators and valuable diagnostic markers in various diseases, such as tumors [15], kidney diseases [16], neurological disorders [17], cardiovascular diseases [18], and diabetes [19]. In reproductive medicine, specific circRNA profiles were identified in ovarian ageing [20], polycystic ovary syndrome (POS) [21,22], and ovarian carcinoma [23,24] and were proved to be aberrantly expressed during pathological changes. These studies have suggested that circRNA plays a role in gene expression and may serve as a potential biomarker and therapeutic target. In animal reproduction, especially in pigs, although it started relatively late, circRNA profiles were identified and compared between different pig breeds [25], during the onset of puberty [26] and during follicular atresia, in our previous studies [27].

The gradually increasing understanding of the regulatory mechanisms of circRNA has revealed that it often acts as a miRNA sponge to regulate target genes and constructs a circRNA–miRNA–gene network. In our study, three rather than one miRNA, termed miR-214-5p/miR-7144-3p/miR-9830-5p, were proved to bind to ssc-circINHA-001. It is reasonable, and has been bioinformatically predicted, that one circRNA may contain more than one miRNA binding site. Recent studies proved multiple sponging functions; for example, circPUM1 promotes ovarian tumorigenesis by sponging miR-615-5p and miR-6753-5p [28]. However, a thorough identification of such miRNA clusters and their downstream targets requires omics data integration. Thus, we made full use of our previous RNA-seq, miRNA-seq, and circRNA-seq results and analyzed possible circRNA–miRNA–mRNA networks in advance. The identification of circINHA-miR-214-5p/miR-7144-3p/miR-9830-5p-INHBA suggested that integrated omics data facilitate the study of circRNA function.

**Figure 7:**
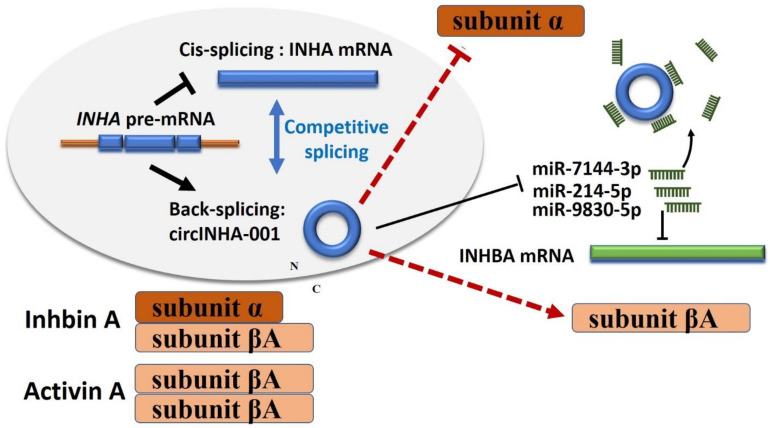
Schematic diagram of ssc-circINHA-001 affecting inhibin and activin balance through competitively binding miR-214-5p/miR-7144-3p/miR-9830-5p in pig GCs. The competitive splicing of circINHA-001 reduced the expression of *INHA* mRNA and α-subunit. Following exportation to the cytoplasm, circINHA can function by competing with *INHBA* mRNA for miR-214-5p/miR-7144-3p/miR-9830-5p interactions, thereby alleviating the posttranscriptional repression of INHBA and thus enhancing βA-subunit. The balance of hormone production may, therefore, tilt toward activin from inhibin and finally induce GC resistance to apoptosis and follicular atresia.

It is worth noticing that, although it is accepted that circRNA functions are generally independent of the circRNA coding gene, our study supports a different conclusion. ssc-circINHA-001 is back-spliced from the inhibin α-subunit coding gene *INHA* and holds binding sites for miR-214-5p/miR-7144-3p/miR-9830-5p. Interestingly, these miRNAs also share one target gene, *INHBA*, which codes for the βA-subunit of inhibin. Theoretically, the product of ssc-circINHA-001 somehow reduced the expression of liner mRNA and protein of the INHA subunit. In addition, increasing circINHA-001 enhanced INHBA subunit expression (Figure 7). In this case, the balance of hormone production would be tilted toward activin, promoting FSH secretion and finally inducing GC resistance to apoptosis and follicular atresia, which nicely agrees with the physiological changes during GC apoptosis and follicular atresia. This inference is also supported by a recent study in bovine, suggesting that *INHBA* could promote the proliferation of GCs, inhibit GC apoptosis, promote the secretion of estrogen and progesterone, and regulate the development of bovine [29] and chicken follicles [30]. In humans, the role of activins and inhibins was valued in pathological research into, for example, cancers and polycystic ovarian syndrome (PCOS). Despite some contradictions in the different cohort studies, it is accepted that the increased expression of activin A contributes to the development of ovarian cancers, breast cancers, and uterine leiomyoma, while inhibin A was reported to be a suppressor of ovarian stromal cell tumors. In PCOS, lower activin A levels and increased inhibin A levels were observed in patients. According to our study, circINHA accelerates activin A formation while inhibiting ncRNAs such as circINHA, providing new insights for therapeutic strategies.

In conclusion, we first identified a new *INHA* gene-coded circRNA, ssc-circINHA-001, which was more highly expressed in healthy than in atretic porcine follicles. ssc-circINHA-001 served as a ceRNA to guarantee the expression of *INHBA* by sponging miR-214-5p, miR-7144-3p, and miR-9830-5p simultaneously, and thus increased the production of activin, with an inhibitory effect on GC apoptosis and follicular atresia. This study demonstrated the existence of a new circINHA–microRNAs–*INHBA* regulatory pathway, and provides new insights into the relationship between a circRNA coding gene and its downstream target gene in ovarian physiological functions, as well as suggestions for the development of new therapeutic methods.

## 4. Materials and Methods

### 4.1. Follicle Collection and Isolation

Porcine ovaries were obtained from healthy, unstimulated 7-month-old commercial gilts from Su Shi slaughterhouse in Huai’an, Jiangsu. The collected porcine ovaries were transferred to the laboratory as soon as possible in physiological saline containing gentamicin sulfate (80 mg/L) at 37 °C. The ovarian tissue was washed and cut with a scalpel in a Petri dish containing PBS. Antral follicles with a diameter of 3–5 mm were isolated using small scissors and forceps [31]. Follicles were divided into HF and AF according to uniform criteria based on follicle appearance, GC density, and progesterone (P4)/estradiol (E2) ratio [32].

### 4.2. Cell Culture and Transfection

The porcine ovaries were washed with sterile physiological saline containing gentamicin sulfate (80 mg/L) at 37 °C until the saline was clear and transparent. GCs were drawn from clear antral follicles with 3–5 mm diameter using a syringe with a 20-gauge needle. GCs were then cultured at 37 °C and 5% CO_2_ in DMEM/F12 medium (Gibco, Carlsbad, CA, USA) containing 10% fetal bovine serum (FBS) (Gibco, Carlsbad, CA, USA), 100 units/mL penicillin, and 100 mg/mL streptomycin. HEK293 cells were maintained under the same conditions in DMEM medium (Sigma, St. Louis, MO, USA). For the FISH assay, the cells were cultured on round coverslips placed in 6-well plates. Inhibitor and mimics of circRNA siRNA and miRNA were synthesized by GenePharma (Shanghai, China) (Supplementary Table S1). Cells were transfected using Lipofectamine^TM^ 3000 (Invitrogen, Carlsbad, CA, USA) and Opti-MEM (Gibco, Carlsbad, CA, USA) according to the manufacturer’s instructions. All cell experiments were done in triplicate.

### 4.3. RNA Preparation and qRT-PCR

Total RNA was extracted using Trizol reagent (Invitrogen, Carlsbad, CA, USA) according to the instructions and qualified by Nanodrop and agarose gel electrophoresis. For the quantitative determination of circRNA, the first-strand cDNA was synthesized using a PrimeScript RT Master Mix (Vazyme, Nanjing, China) kit. qPCR was performed by the ABI StepOne system, Applied Biosystems, Carlsbad, CA, USA) using SYBR Premix Ex Taq (Takara, Dalian, China), according to the manufacturer’s instructions, with GAPDH as the internal reference. For RNase-R-treated samples, GAPDH without RNase R treatment was considered as an internal reference for all samples. For miRNA quantification, reverse transcription was performed with the BioTek miRNA cDNA first-strand synthesis kit (BioTeke, Wuxi, China). Then, detection was performed with the BioTek miRNA fluorescence quantitative detection kit (BioTeke, Wuxi, China), with U6 as the internal reference. The relevant primer information is listed in Supplementary Table S2.

### 4.4. RNase R Treatment and RT-PCR Product Sequencing 

The extracted RNA of each GC sample was divided into two equal aliquots. One aliquot was directly subjected to reverse transcription, while the other was treated with RNase R (Epicentre, Madison, WI, USA) before reverse transcription. The 20 μL digestive system included 5 μg RNA, 2 μL 10× Reaction Buffer, 15 U RNase R(20 U/μL), and RNase-Free water. The reaction was performed at 37 °C for 15 min and then at 70 °C for 10 min, using SYBR Premix Ex Taq (Takara, Dalian, China), according to the manufacturer’s instructions. The sequence of the PCR products was verified by agarose gel electrophoresis and Sanger sequencing (Bioengineering, Shanghai, China).

### 4.5. Dual Luciferase Activity Assay

ssc-circINHA-001 sequences containing two wild-type (WT1, WT2) or mutant (MUT1 MUT2) binding sites for miR-214-5p, two wild-type (WT1, WT2) or mutant (MUT1 MUT2) binding sites for miR-9830-5p, and three wild-type (WT1, WT2, WT3) or mutant (MUT1, MUT2, MUT3) binding sites for miR-7144-3p were constructed. The pmirGLO Dual-Luciferase miRNA Target Expression Vector (Promega Corporation, Madison, WI, USA) containing the sequence of each circRNA binding sites and the corresponding miRNA mimics were synthesized by Qingke Biotechnology Co., Nanjing, China. The binding sites and mutated sequences are listed in the Supplementary Tables S3–S6.

### 4.6. Western Blotting

GCs were collected 48 h after transfection, and RIPA buffer (Bioworld, Nanjing, China) containing 1% PMSF (Bioworld, Nanjing, China) was added to lyse the cells. Protein concentration as determined by a BCA determination kit (Beyotime, Shanghai, China). Protein samples were separated by SDS-PAGE according to our previous description [33]. The antibodies used in this study were anti-tubulin (TUBB) (diluted 1:1000, #6181S, Cell Signaling Technology, Boston, MA, USA) and anti-Cleaved Caspase-3 (C-CASP3) (diluted 1:1000, #9664S, Cell Signaling Technology, Boston, MA, USA), and the secondary antibody (diluted 1:2000, SA00001-2, Proteintech Group, Rosemont, IL, USA). Protein levels were detected with an ECL Plus reagent (Promega, Madison, WI, USA) and analyzed using ImageJ software (v1.8.0). Each experiment was performed three times.

### 4.7. FISH

Labelled probes were specifically synthesized for ssc-circINHA-001, miR-214-5p, miR-7144-3p, and miR-9830-5p by Wuhan Seville Biotech, and DAPI was used to stain the cell nuclei. GCs were cultured on coverslips, fixed in 4% paraformaldehyde (containing DEPC) for 20 min, washed three times while shaking with PBS (pH 7.4), and proteinase K (20 µg/mL) was finally added for 5 min for digestion [27]. All procedures were conducted according to the manufacturer’s instructions (Sevicebio, Wuhan, China). Fluorescent images were acquired using a Nikon upright fluorescence microscope (Nikon DS-U3, Tokyo, Japan). Each experiment was performed three times. The probe sequences are shown in Supplementary Table S7.

### 4.8. Apoptosis Assay

GCs apoptosis was assessed by the Annexin V-FITC/PI staining assay (Vazyme, Nanjing, China) according to the manufacturer’s protocol and our previous description [27]. The percentage of apoptotic cells was determined by flow cytometry (Becton Dickinson FACS Calibur, Franklin Lakes, NJ, USA). The data were analyzed using FlowJo v7.6 software (Stanford University, Stanford, CA, USA).

### 4.9. ELISA Assay

The expression level of INHBA in GC cultures was detected using the INHBA ELISA assay kit according to the manufacturer’s instructions (Jianglaibio, Shanghai, China). The OD value of each well was detected at 450 nm by a plate reader (Multiskan GO, Thermo Scientific) and analyzed by the supplied program.

### 4.10. Statistical Analysis

GraphPad Prism 5 software was used to perform statistical analyses. Two-tailed Student’s *t*-tests were used to evaluate significance when the two groups were compared. All data are presented as the mean ± SEM. *p*-values of <0.05 (*) and 0.01 (**) were considered to indicate significant and highly significant differences, respectively.

## Supplementary Materials

The following are available online at https://www.mdpi.com/article/10.3390/ijms22179113/s1. Table S1. Oligonucleotide sequences; Table S2. Information on primers; Table S3. Binding sites of miR-214-5p on ssc-circINHA-001; Table S4. Binding sites of miR-7144-3p on ssc-circINHA-E1; Table S5. Binding sites of ssc-circINHA-001 and miR-9830-5p; Table S6. Binding sites of miR-214-5P/miR-7144-3p/miR-9830-5p on INHBA 3′-UTR; Table S7. FISH probe sequences; Figure S1. Bioinformatic analysis prediction of interactions among ssc-circINHA-001 and microRNAs done by Bibiserv; Figure S2. The prediction and screening of potential target genes for miR-214-5p, miR-7144-3p and miR-9830-5p.
