# Supplementary material for: circRNA-Mediated Inhibin–Activin Balance Regulation in Ovarian Granulosa Cell Apoptosis and Follicular Atresia
Source: Int J Mol Sci. 2021 Aug 24;22(17):9113. doi: 10.3390/ijms22179113 (PMC8431694; doi:10.3390/ijms22179113)
Supplement: Supplementary file 1 [file ijms-22-09113-s001.zip › ijms-1349903-supplementary.pdf]

Supplementary Table S1. Oligonucleotide sequences

Name	Sequence
ssc-circINHA-001-siRNA	Sense: AGACUCCUGUCCCCAACCGTT
	Anti-sense: CGGUUGGGGACAGGAGUCUTT
INHBA-siRNA	Sense: GGGCGGAAAUGAAUGAACUTT
	Anti-sense: AGUUCAUUCAUUUCCGCCCTT
miR-214-5p mimics	Sense: UGCCUGUCUACACUUGCUGUGC
	Anti-sense: ACAGCAAGUGUAGACAGGCAUU
miR-9830-5p mimics	Sense: GUCUGGAAAGGUCGGAUGAGU
	Anti-sense: UCAUCCGACCUUUCCAGACUU
miR-7144-3p mimics	Sense: GCUCCUUGUCCCGAGACCGCGA
	Anti-sense: GCGGUCUCGGGACAAGGAGCUU
miR-214-5p inhibitor	Sense: GCACAGCAAGUGUAGACAGGCA
miR-9830-5p inhibitor	Sense: ACUCAUCCGACCUUUCCAGAC
miR-7144-3p inhibitor	Sense: UCGCGGUCUCGGGACAAGGAGC
mimics/si NC	Sense: UUCUCCGAACGUGUCACGUTT
	Anti-sense: ACGUGACACGUUCGGAGAATT
inhibitor NC	Sense: CAGUACUUUUGUGUAGUACAA

Supplementary Table S2. The information of primers

Gene	Acc.No.	Primer （5'- 3'）	function
ssc-circINHA-E1	ENSSSCE 00000168706	F:TGGTTGAAAGGAGACCCTGAAG<br>R:CCCTGCTCTATACTTAGCCTGGA	PCR&qRT-PCR
GAPDH	AF017079	F: GGACTCATGACCACGGTCCAT<br>R: TCAGATCCACAACCGACACGT	qRT-PCR
miR-214-5p	MN753348.1	F:UGCCUGUCUACACUUGCUGUGC<br>R: CAGCCACAAAAGAGCACAAT	qRT-PCR
miR-9830-5p	NR_128517.1	F:GUCUGGAAAGGUCGGAUGAGU<br>R: CAGCCACAAAAGAGCACAAT	qRT-PCR
miR-7144-3p	MN753827.1	F:GCUCCUUGUCCCGAGACCGCGA<br>R: CAGCCACAAAAGAGCACAAT	qRT-PCR
U6	EU520423.1	F:CGCTTCGGCAGCACATATAC<br>R:TTCACGAATTTGCGTGTCAT	qRT-PCR
INHBA	NM_214028.1	F:GAGGGCGGAAATGAATGAAC<br>R:GTTGAAAGAGACGGATGGAGACT	qRT-PCR

Supplementary Table S3.

The binding sites of miR-214-5p on ssc-circINHA-001

Name	Region	Binding site sequence
ssc-circINHA-001-WT	102~109…117~136nt	...CCCAGACAGGC...GGCTAAGTATAGAGCAGGGGGGA...
ssc-circINHA-001-MUT-1	102~109…117~136nt	...CCCGAGTGAAT...GGCTAAGTATAGAGCAGGGGGGA...
ssc-circINHA-001- MUT-2	102~109…117~136nt	...CCCAGACAGGC...AAATCGGACACGAGATGAAGAGGA...
ssc-circINHA-001- MUT-3	102~109…117~136nt	...CCCGAGTGAAT...AAATCGGACACGAGATGAAGAGGA...

Note: 1nt represents pig chromosome 12 121581344

Supplementary Table S4.

The binding sites of miR-7144-3p on ssc-circINHA-E1

Name	Region	Binding site sequence
ssc-circINHA-001-WT-1	90~96nt	...GGAGGACAAGAGC...
ssc-circINHA-001-WT-2	130~137nt	...AGAGCAGGGGGGAC...
ssc-circINHA-001-WT-3	223~228nt	...TGAAAGGAGACC...
ssc-circINHA-001- MUT -1	90~96nt	...GGAAAGTGGAAGC...
ssc-circINHA-001- MUT -2	130~137nt	...AGAATGAAAAAGAC...
ssc-circINHA-001- MUT -3	223~228nt	...TGACCTTCTACC...

Note: 1nt represents pig chromosome 12 121581344

Supplementary Table S5.

The binding sites of ssc-circINHA-001 and miR-9830-5p

Name	Region	Binding site sequence
ssc-circINHA-001-WT	251~270nt…299~311nt	...CTGGCTTGTCCTGTCTTCCTGAC...CCAGCTTCCAGACTCC…
ssc-circINHA-001-MUT-1	251~270nt…299~311nt	...CTGTAGGTGAAGGTCGGAAGTCA...CCAGCTTCCAGACTCC...
ssc-circINHA-001- MUT-2	251~270nt…299~311nt	...CTGGCTTGTCCTGTCTTCCTGAC...AAATAGGAACTCATCC...
ssc-circINHA-001- MUT-3	251~270nt…299~311nt	...CTGTAGGTGAAGGTCGGAAGTCA...AAATAGGAACTCATCC...

Note: 1nt represents pig chromosome 12 121581344

Supplementary Table S6. The binding sites of miR-214-5P/miR-7144-3p/miR-9830-5p on INHBA 3'-UTR

Name	miRNA	Region	Binding site sequence
INHBA-3'UTR-WT-1	miR-214-5p	731~737nt	TTCGTGATGGGCCTGT
INHBA-3'UTR- MUT -1	miR-214-5p	731~737nt	TTCGTAACCCAACTGT
INHBA-3'UTR-WT-2	miR-7144-3p	239~250nt	GGGGCGGGGCGGGGGGGGAC
INHBA-3'UTR- MUT -2	miR-7144-3p	239~250nt	GGGGAAAAAAAAAAACCGGAC
INHBA-3'UTR-WT-3	miR-9830-5p	605~613nt	ACTTTTCTGAGACAAAG
INHBA-3'UTR- MUT -3	miR-9830-5p	605~613nt	ACTTACCCCCCCCAAAG

Note: 1nt represents 1309 of INHBA mRNA

Supplementary Table S7. FISH probe sequences

Name	Sequence	Probe type
ssc-circINHA-001	5’-CGGTTGGGGACAGGAGTCTGGAAGCTG-3’	FAM
ssc-circINHA-001	5’-GGCGGTTGGGGACAGGAGTCTGGAAGC-3’	DIG
miR-214-5p	5’-GCACAGCAAGTGTAGACAGGCA-3’	DIG
miR-9830-5p	5’-ACTCATCCGACCTTTCCAGAC-3’	DIG
miR-7144-3	5’-TCGCGGTCTCGGGACAAGGAGC-3’	DIG
